# Changes in blood biochemical parameters in highly productive cows with ketosis

**DOI:** 10.14202/vetworld.2024.1130-1138

**Published:** 2024-05-17

**Authors:** Yelena Il, Dmitrii Il, Mikhail Zabolotnykh, Inna Savenkova, Kulsara Nurzhanova, Daniyar Zhantleuov, Bolatpek Kozhebayev, Balnur Akhmetova, Kaliya Satiyeva, Lailim Kurmangali

**Affiliations:** 1 Department of Food Security, Agrotechnological Faculty, Manash Kozybaev North Kazakhstan University, Petropavlovsk, Kazakhstan; 2 Department of Veterinary and Sanitary Expertise of Livestock Product and Hygiene of Farm Animals, Faculty of Veterinary Medicine, P.A. Stolypin Omsk State Agrarian University, Omsk, Russian Federation; 3 Department of Agronomy and Forestry, Agrotechnological Faculty, Manash Kozybaev North Kazakhstan University, Petropavlovsk, Kazakhstan; 4 Department of Agriculture and Bioresources, Faculty of Veterinary Medicine and Agricultural Management, Shakarim University of Semey, Semey, Kazakhstan; 5 Department of Livestock, North Kazakhstan Research Institute of Agriculture, Beskol, Kazakhstan

**Keywords:** biochemical composition of blood, ketone bodies, liver dystrophy, metabolism, phospholipids

## Abstract

**Background and Aim:**

Biochemical blood testing is the main diagnostic indicator of the clinical condition of highly productive animals and a method of determining changes in metabolic disorders. This study focuses on metabolic changes (ketosis), which are of the utmost importance in the assessment of the health status of animals, as well as differences in intergroup characteristics. The main focus of this study is to demonstrate the influence of subclinical ketosis in highly productive cows on changes in biochemical blood parameters during different physiological periods to further prevent this disease, adjust feeding rations, and prevent premature culling of animals. This study aimed to evaluate and establish changes in the biochemical status dynamics of highly productive cows with metabolic disorders in an industrial livestock complex.

**Materials and Methods:**

Blood samples were systematically collected from highly productive cows of the Simmental breed (n = 60) and served as the primary material for subsequent analyses. Each methodological step was designed to ensure evaluation of the metabolic changes associated with post-calving adjustments in highly productive dairy cows. This study employed a comprehensive approach integrating clinical assessments, laboratory analyses, biochemical evaluations, instrumental measurements, and statistical analyses.

**Results:**

A biochemical blood test showed that the number of ketone bodies in the experimental group exceeded the norm, varied depending on the physiological state of the animals, and ranged from 0.89 to 1.45 mmol/L. At 10 days after calving, the highest indicator was 1.45 ± 0.05 mmol/L. This indicator was 1.05 mmol/L higher than that in the control group and exceeded the norm by 0.95.

**Conclusion:**

Excess ketone bodies in the blood of animals led to accumulation in urine and milk, indicating a disturbance in metabolic processes in the body and a decrease in the quality of animal husbandry products. The sample size and the focus on a single breed from one geographical location may limit the generalizability of the findings. Further research should explore the mechanistic bases of ketosis development, potentially integrating genomic and proteomic approaches to understand the genetic predispositions and molecular pathways involved.

## Introduction

The intensification of industrial animal husbandry necessarily leads to excessive functional stress on the animal’s body, which in some cases leads to pathologies. This situation creates conditions for metabolic diseases such as ketosis, acidosis, and glycocalcemia [1–3]. Ketosis is a widespread metabolic disease in dairy cows [4]. It is polyethological and is mainly associated with the digestive peculiarities of ruminants. The main causes of ketosis are errors in the feeding of cows after calving, such as unbalanced diets and their inconsistency with the physiological needs of animals, carbohydrate deficiency during the period of maximum milk production, excess protein, and concentrated feed in a diet lacking hay and coarse feed, as well as lack of exercise and stress exposure.

The study of the mechanisms underlying the development of metabolic disorders in highly productive animals makes it possible to increase the life of animals, increase the productivity of cows, and obtain high-quality products. The quality of milk and its products directly depends on the health and metabolic processes in the cow’s body. Violation of feeding and maintenance regimes, stress, and violation of the milking regime of cows lead to metabolic disorders, which can result in a decrease in the quality of milk, as well as causing significant economic damage to livestock farming. Today, one of the important tasks in animal husbandry and veterinary medicine is the creation of a highly productive, sustainable herd with a stable metabolic rate [5, 6].

Metabolic diseases are most often detected in animals during the peak physiological stress of the body, including pregnancy, calving, lactation, and growth. It should be noted that lipid metabolism disorders are often accompanied by the emergence of a ketogenic state and subsequent development of ketosis [7, 8].

Ketosis is accompanied by a disturbance of the main metabolic processes. Dystrophic changes in internal organs, accumulation of ketone bodies in tissues, blood, urine, and milk, changes in the biochemical composition of blood, such as hypoglycemia or hypocalcemia, a decrease in alkaline reserve, and a decrease in the level of the main indicators of erythropoiesis [5, 6] are characteristic signs.

In ketosis, the degree of metabolic disorders can vary significantly depending on the stage of the technological period. It is important to study the metabolism and changes in the biochemical parameters of blood during different periods in cows. High productivity in cows is associated with intensive metabolism. To optimize active metabolic processes, it is necessary for animals to receive an optimal amount of all normalized nutrients from their diet. In practice, complex insufficiency of several elements (energy, protein, carbohydrates, and minerals) is most often observed in highly productive herds, which complicates the realization of the genetic potential of animals [9–11]. Therefore, consideration of metabolic features and their timely correction is one way to increase the efficiency of dairy cattle breeding [7, 8].

In this study, we meticulously differentiated between ketosis as a potential bidirectional predictor and its role as an outcome variable in highly productive cows with metabolic disorders. Our analysis delineates the cause-and-effect relationship by examining the onset of ketosis and its impact on blood biochemical parameters. Specifically, we explore how elevated ketone bodies not only serve as an indicator of metabolic stress but also contribute to further biochemical alterations, signifying a complex interplay between causes and effects. This distinction is crucial for understanding the pathophysiological mechanisms of ketosis and guiding our investigation into the sequential biochemical changes that characterize ketosis.

This study aimed to investigate the dynamics of changes in biochemical blood parameters during subclinical ketosis in highly productive Simmental cows at different physiological periods.

## Materials and Methods

### Ethical approval

The study protocol was discussed and approved at the meeting of the local ethical commission of the Agrotechnological Faculty of North Kazakhstan University named after Manash Kozybayev on September 24, 2020.

### Study period and location

The study was conducted from September 2020 to May 2023 under the production conditions of one of the dairy farms in the North Kazakhstan region, with a high productivity of Simmental breed animals. Laboratory blood tests of experimental animals were performed at the North Kazakhstan University, named after M. Kozybayev, Private Animal Science Laboratory.

### Experimental design

Our study meticulously measured blood levels of beta-hydroxybutyrate, acetoacetate, and acetone to track ketosis progression. In addition, changes in cholesterol, triglycerides, and other critical biochemical parameters, such as glucose and calcium levels, were evaluated. By employing these indicators, we were able to establish a clear relationship between ketosis and its direct impact on post-second calving biochemical status of high-yield Simmental cows.

### Sampling

To conduct the study on the principle of pairs of analogs, highly productive cows of the Simmental breed were selected, taking into account the physiological state, in 60 heads from the total livestock on a dairy farm of 400 heads. Most cows have two calvings, an average live weight of 600–650 kg, and an average annual milk yield of 6–7 thousand kg per lactation.

Experimental and clinical studies were conducted by forming experimental and control groups (healthy animals and animals with metabolic disorders) of animals. In accordance with the results of urine samples with sodium nitroprusside for the presence of ketone bodies using Ketoglyuk test strips (Biosensor company, Russia), the concentration of ketone bodies in the blood was studied using a FreeStyle Optium glucometer (Abbott company, USA) using FreeStyle Optium test strips adapted for measuring hydroxybutyric acid, as the most stable fraction of ketone bodies.

Studies of urine and blood samples for the presence of ketone bodies from 60 selected cows revealed that the average amount of ketone bodies in urine and blood was higher than physiological norms in 22 (36.6%) and normal in the remaining 38 animals (63.4%). This confirms the fact that 22 animals had metabolic disorders with subclinical ketosis, which latently progressed. On the basis of the results of tests for the presence of ketone bodies in the urine and blood of the selected 60 animals, two groups of 20 animals each were formed: the first (control) group comprised clinically healthy animals and the second (experimental) group comprised patients with subclinical ketosis.

The conditions for keeping, feeding, and caring of the animals were the same. In the course of the experiment, cows received rations with feed of their own production, including grain haylage, corn silage, coarse feed, and compound feed for cattle produced in the feed processing room. In the course of the study, we monitored animal feeding according to the diet, depending on the physiological state of the animals, as well as the nutrient content of the feed for the experimental and control groups [12, 13]. Manure cleaning was performed using scraper conveyors of the TSN type (Krasny Press plant, Russia), and watering was performed using automatic drinking fountains AP-1A (Himagromash LLP, Kazakhstan).

The selected animals were divided into two groups of 20 heads: the first (control) group included clinically healthy animals and the second (experimental) group included animals with subclinical ketosis.

### Biochemical analysis

Biochemical parameters of cows’ blood were tested according to 14 indicators: total ketone bodies concentration (TKBC) and their fractions (b-hydroxybutyric acid [BH] and acetone with acetoacetic acid [AcAc]), total protein, protein fractions (albumins, α-, β-, γ-globulins), cholesterol, GluOx (glucose), urea, reserve alkalinity, phospholipids, and triglycerides in animal.

The biochemical status of the studied groups was assessed 4 times each year: 2 months before calving, a month before calving, 10 days after calving, and a month after calving. Blood samples from 40 highly productive Simmental cows were selected for this study. Blood for biochemical studies was collected from the tail vein in the morning hours before feeding in 9 mL vacuum tubes with a coagulation activator (Greiner Bio-One, Austria) using 1.25 × 38 mm 18G 1.5 double-sided needles for a single intake and a standard non-sterile vacuum holder in compliance with the rules of asepsis and antiseptics. Blood serum was examined without signs of hemolysis, lacteous serum, and icterus.

Biochemical blood tests were performed on a BioChem SA semi-automatic biochemical analyzer with a built-in flow cuvette (High Technology, Inc., USA). Total protein, albumins, urea, glucose, cholesterol, and triglycerides in the serum and plasma of animals were determined using a BioChem SA semi-automatic analyzer with sets of liquid, ready-to-use HTI biochemical reagents. The total concentration of ketone bodies and their fractions was determined by iodometric analysis.

### Statistical analysis

Statistical processing of the obtained results was performed using Microsoft Excel (Microsoft Corporation, USA) and biometric methods (variational-statistical method for processing experimental data). Two-factor analysis of variance was used to determine the presence of statistically significant differences between the groups. Microsoft Excel and IBM Statistical Package for the Social Sciences Statistics 22 (IBM Corp., NY, USA) were used for data analysis. All data were statistically processed and are presented as the mean (M) and error of the mean (m) [14].

In the course of this research, it is necessary to demonstrate that biochemical blood parameters in animals change statistically significantly during different physiological periods during subclinical ketosis.

## Results

The blood in the body plays a significant role in metabolism, which is carried out through it. When analyzing the obtained data, we found a change in blood biochemical parameters in healthy and subclinical ketosis cows, depending on the calving state.

Table-1 shows the levels of ketone bodies in the studied groups.

**Table 1:** Indicators of TKBC and their fractions in the blood of animals (M ± m) (n = 20).

Indicator	Time of the study			

	2 months before calving	1 month before calving	10 days after calving	1 month after calving
Control group (healthy, n = 20)				
TKBC, mmol/L	0.48 ± 0.02	0.50 ± 0.01	0.40 ± 0.03	0.37 ± 0.02
AcAc, mmol/L	0.15 ± 0.02	0.27 ± 0.03	0.20 ± 0.03	0.17 ± 0.02
BH, mmol/L	0.51 ± 0.05	0.58 ± 0.03	0.54 ± 0.07	0.60 ± 0.09
Experimental group (animals with subclinical ketosis, n = 20)				
TKBC, mmol/L	0.89 ± 0.04	1.0 ± 0.02	1.45 ± 0.05	1.07 ± 0.03
AcAc, mmol/L	0.42 ± 0.04	0.87 ± 0.02	0.78 ± 0.05	1.05 ± 0.06
BH, mmol/L	0.98 ± 0.09	1.11 ± 0.12	1.00 ± 0.05	1.5 ± 0.07

p ≤ 0.001, TKBC=Total ketone bodies concentration, AcAc=Acetoacetic acid, BH=b-hydroxybutyric acid, M=Mean, m=Error of the mean

As a result of the obtained data, it should be noted that the TKBC concentration in the experimental group was significantly higher than that in the control group during the entire experiment: 0.41 mmol/L, 0.5 mmol/L (p ≤ 0.001), 1.05 mmol/L (p ≤ 0.001), and 0.7 mmol/L (p ≤ 0.001) before calving in the first, second, and third studies, respectively.

According to the results of the analysis for ketone bodies, the animals of the experimental group had subclinical ketosis because the level of ketone bodies in their blood ranged from 0.89 to 1.45 mmol/L. The entry of large quantities of fatty acids into the mitochondria of hepatocytes leads to the formation of acetyl-coenzyme A molecules, which bypass the tricarboxylic acid cycle and proceed to the synthesis of acetoacetic and BH acids. All ketone bodies are derivatives of AcAc.

Table-1 presents the results of the study of TKBC and their fractions (AcAc and BH) in the blood of cows.

A study on the presence of AcAc in the blood of highly productive animals showed that 2 months before calving, the level of AcAc in the blood of the experimental group was 0.26 higher than that in the blood of the control group (p < 0.001). After calving, the concentration of AcAc increased. It was 0.58 mmol/L (p ≤ 0.001) higher in the experimental group 10 days after calving than in the control group. A month after calving in the experimental group, the indicator was 1.05 ± 0.05, which was 0.27 mmol/L higher than that in the second study.

In the control group, the concentration of AcAc was within physiological limits (no increase was observed) throughout the experiment, with an average of 0.21 ± 0.03 mmol/L before calving and 0.18 ± 0.2 mmol/L after calving.

Analysis of the results of the studied groups of cows showed that throughout the entire period of the study, the indicators were higher in the experimental group than in the control group before calving by 0.27 mmol/L (p ≤ 0.001), 0.60 mmol/L (p ≤ 0.001), 0.58 mmol/L (p < 0.001), and 0.88 mmol/L (p < 0.001) in the first, second, and third studies, respectively, compared with the control group before calving. Elevated levels of AcAc indicate metabolic disorders in animals and accumulation of ketone bodies in tissues and blood.

Throughout the entire study period, the BH acid index in the experimental group exceeded the norm. 2 months before calving, the indicator in the experimental group was 85.4% (p ≤ 0.001) higher than that in the control group, 0.9 mmol/L (p ≤ 0.001) in the fourth study (a month after calving), 0.53 mmol/L (p ≤ 0.01) in the second study (a month before calving), and 0.43 mmol/L p < 0.01 in the third study (10 days after calving).

Table-2 shows the concentrations of triglycerides, cholesterol, and phospholipids in the blood of cows. As shown in Table-2, the dynamics of the changes in cholesterol concentration in all the studied groups were within physiological values. Throughout the entire study period, the level of this indicator in the blood of cows in the experimental group exceeded the same indicator in control group. Therefore, before calving in the first study, the cholesterol level in the blood of cows of the experimental group exceeded that in the control group by 1.94 mmol/L.

**Table 2:** Concentration of triglycerides, cholesterol, and phospholipids in the blood of cows (mmol/L, M ± m, n = 20).

Indicator	Time of the study			

	2 months before calving	1 month before calving	10 days after calving	1 month after calving
Control group (healthy, n = 20)				
Triglycerides	0.45 ± 0.03***	0.40 ± 0.06*	0.32 ± 0.05*	0.38 ± 0.02***
Cholesterol	1.84 ± 0.09***	2.89 ± 0.13***	3.32 ± 0.15***	2.69 ± 0.11***
Phospholipids	2.71 ± 0.09***	2.62 ± 0.12***	2.36 ± 0.07***	2.47 ± 0.11***
Experimental group (animals with subclinical ketosis, n = 20)				
Triglycerides	0.69 ± 0.06***	0.61 ± 0.09*	0.48 ± 0.05*	0.65 ± 0.04***
Cholesterol	3.78 ± 0.18***	4.02 ± 0.21***	4.26 ± 0.14***	3.57 ± 0.19***
Phospholipids	2.09 ± 0.16***	1.83 ± 0.10***	1.69 ± 0.09***	1.95 ± 0.11***

* p ≤ 0.05,

*** p ≤ 0.001, M=Mean, m=Error of the mean

The cholesterol concentration increased significantly in the blood of cows of the experimental group 10 days after calving and approached the maximum physiological value of 4.26 ±0.14 mmol/L, which was 0.24 mmol/L and 0.48 mmol/L compared with the second study (1 month before calving) at the level of statistical significance (p ≤ 0.002).

A month after calving, the cholesterol level in the blood of cows of the experimental group decreased significantly. It amounted to 3.57 ± 0.19 mol/L, which, in turn, compared with the third study (10 days after calving), was lower by 0.69, compared with the first study by 0.21, and with the second study (for a month before calving) by 0.45. At 10 days after calving, the cholesterol level in the control group was 3.32 ± 0.15 mol/L, which was 1.48 mmol/L more than a month before calving, but it was within the normal range. In the fourth study, the indicator was 0.88 mmol/L higher in the experimental group compared to the control group. The triglyceride levels in the blood of the experimental and control groups are presented in Table-2.

According to Table-2, the level of triglycerides in the group of healthy animals was within the physiological norm; however, there was a gradual decrease in triglyceride levels. At 10 days after calving, triglyceride levels decreased by 28% or 0.13 mmol/L compared with that 1 month before calving. A month after the calving observed this indicator increased by 0.06 mmol/L and equaled 0.38 ± 0.02 mol/L.

The level of triglycerides in the blood of the experimental group was higher than that in the control group throughout the study period. The triglyceride content in the group of sick animals exceeded the norm by 15% 2 months before and 10 days after calving, which decreased by 21% compared to the previous study.

Thirty days after calving during lactation, there was a significant increase in triglyceride concentrations in the blood serum of the experimental and control groups. In the control group, the indicator amounted to 0.38 ± 0.02 mmol/L and was within the normal range. In the experimental group, the indicator value was 0.65 ± 0.04, which was 0.05 or 8% higher than normal, respectively.

Table-2 indicates that 2 months before calving in the experimental group, the level of this indicator was the highest at 2.09 ± 0.16 mmol/L for the entire study period. Subsequently, a decrease in the concentration of phospholipids in the blood of animals of the experimental group can be noted. The tendency of this indicator to increase after calving in the control and experimental groups reflects the formation of metabolic processes and the activation of physiological reactions of the body during this period. In the fourth study, the average values in the experimental group of cows were 27% lower than those in the control group (p ≤ 0.001).

Table-3 shows the concentrations of glucose, alkaline reserves, and urea.

**Table 3:** Concentration of glucose, alkaline reserve, and urea in the blood of cows (mmol/L, M ± m, n = 20).

Indicator	Time of the study			

	2 months before calving	1 month before calving	10 days after calving	1 month after calving
Control group (healthy, n=20)				
Glucose	3.47 ± 0.23***	4.04 ± 0.18***	3.86 ± 0.17***	4.11 ± 0.14***
Alkaline reserve level	19.38 ± 1.15*	20.02 ± 1.08*	18.50 ± 1.00*	19.79 ± 1.12*
Urea	4.8 ± 0.25***	4.23 ± 0.30***	4.52 ± 0.28***	3.77 ± 0.33***
Experimental group (animals with subclinical ketosis, n = 20)				
Glucose	2.22 ± 0.16***	2.13 ± 0.22***	2.00 ± 0.19***	2.12 ± 0.20***
Alkaline reserve level	16.59 ± 1.17*	16.96 ± 1.20*	16.01 ± 1.08*	17.07 ± 1.15*
Urea	2.23 ± 0.30***	2.58 ± 0.27***	1.59 ± 0.45***	2.05 ± 0.40***

* p ≤ 0.05,

*** p ≤ 0.001, M=Mean, m=Error of the mean

The data presented in Table-3 indicate that the glucose content in the blood of both groups had different dynamics during the entire experimental period. In the experimental group of animals, the glucose concentration was maintained at a minimum physiological level throughout the experiment. A month before calving, the glucose content in both groups decreased, but in the control group, this indicator amounted to 4.04 ± 0.18 mmol/L, within the physiological norm. However, the dynamics of the decrease were significantly higher in the experimental group than in the control group, and this indicator in the experimental group was 2.13 ± 0.22, which was 1.91 below the norm.

Based on the studies conducted to determine the main biochemical indicator of blood glucose in the body of animals, there was an irrational expenditure of energy on metabolic processes and the formation of milk, which resulted in a decrease in blood glucose levels immediately after calving. Furthermore, in the blood of cows with ketosis, the content of this indicator was 1.86 mmol/L lower than that in the blood of clinically healthy cows during this period.

We observed an increase in blood glucose levels in both groups a month after calving. Thus, in the experimental group, this indicator increased by 0.7 mmol/L compared to the third study and by 0.2 mmol/L in the second study. Blood glucose levels in highly productive animals of the control group were maintained within relatively constant limits throughout the experiment due to the complex mechanisms of neurohumoral regulation, which primarily affect liver function.

According to our results, the concentration of urea in the blood of animals in the control group was within the physiological norm, and there were no significant changes in the dynamics of indicators during different periods of the study. Urea concentrations in the blood of cows in the control group ranged from 3.77 ± 0.33 mmol/L to 4.8±0.25 mmol/L and did not deviate from the norm.

In the subclinical ketosis group, there was a decrease in the level of urea from the normal level. At 10 days after calving, the lowest indicator amounted to 1.59 ± 0.45 mmol/L, which was 1.91 below the norm.

According to the results of the analysis of the urea content in the blood, deviations from the norms of energy and protein nutrition, as well as the provision of energy to cows, were observed. Considering that the diets were balanced in energy and protein, it is likely that the decrease in urea concentration in the blood was caused by the disturbance of liver function.

Table-3 shows the alkaline reserves. According to Table-3, 2 months before calving, the level of alkaline reserve in the blood of cows of the experimental group was 2.79 mmol/L lower than that of the control group. The data obtained indicate that the dynamics of decline during all the studied periods proceeded faster and more intensively in the experimental group than in the control group. Thus, the alkaline reserve content in the blood a month before calving was 0.37 mmol/L in the experimental group and 0.64 mmol/L in the control group compared to the level of the first study.

The decrease in the alkaline reserve in the blood of experimental cows compared to the third study was 6%, whereas it was only 8.3% in the blood of the control group. Despite the decrease in the alkaline reserve values in the blood of cows with ketosis compared with clinically healthy cows, no large and significant differences between the groups were found during the entire study.

On the basis of these findings, the level of alkaline reserve by the past month of pregnancy, as well as during intensive milking, has a more pronounced dynamics, is also prone to decrease, and has lower values in cows with ketosis compared to clinically healthy cows.

The total protein indicators are shown in Table-4. According to Table-4, before calving, the level of total protein in the blood of the experimental group was 6.3 times higher than that in the control group; however, a month before calving, the concentration of total protein in the blood of cows with ketosis decreased slightly. On the other hand, in the control group, the level of total protein increased by 7% compared to the initial value. In the second study, the average values of the experimental and control groups differed significantly by 12% (p = 0.001) but were within the normal range. Table-4 presents the protein content and its fractions in the blood.

**Table 4:** Protein content and its fractions in the blood of the studied cows (M ± m, n = 40).

Indicator	Time of the study			

	2 months before calving	1 month before calving	10 days after calving	1 month after calving
Control group (healthy, n = 20)				
Total protein, g/L	74.8 ± 1.90**	79.6 ± 2.21***	68.6 ± 2.47***	70.9 ± 2.33**
Albumins, %	38.74 ± 1.25*	35.03 ± 1.20**	39.89 ± 1.18*	40.63 ± 1.21*
Alpha globulins, %	18.1 ± 0.50***	16.89 ± 1.30***	13.17 ± 0.90**	17.06 ± 0.40***
Beta globulins, %	15.1 ± 1.16***	20.20 ± 1.10***	16.25 ± 1.25**	14.23 ± 1.09*
Gamma globulins, %	29.63 ± 1.49**	37.0 ± 1.62***	34.06 ± 1.50**	30.55 ± 1.32**
Experimental group (animals with subclinical ketosis, n = 20)				
Total protein, g/L	81.1 ± 2.00**	69.84 ± 2.27***	75.9 ± 2.35***	79.8 ± 2.31**
Albumins, %	33.85 ± 1.62*	30.11 ± 1.32	35.83 ± 1.22*	37.01 ± 1.10*
Alpha-globulins, %	12.60 ± 0.8***	10.42 ± 1.02***	9.96 ± 0.77**	12.52 ± 0.68***
Beta-globulins, %	21.4 ± 1.02***	27.32 ± 1.20***	20.37 ± 1.17**	17.66 ± 1.24*
Gamma-globulins, %	35.70 ± 1.78**	45.03 ± 1.87***	39.61 ± 1.74**	35.01 ± 1.66**

* p ≤ 0.05,

** p ≤ 0.01,

*** p ≤ 0.001, M=Mean, m=Error of the mean

The albumin level in the experimental group 2 months before calving was below the extreme physiological parameters by 7% and 1 month before calving by 17%. At 10 days after calving, it was within the normal range and amounted to 35.83 ± 1.22.

Our data give us reason to conclude that albumin concentrations were significantly lower in the experimental group than in the control group in the first study by 12.6% (p < 0.05), in the second study by 16.4% (p < 0.01), and in the fourth study by 8.2% (p < 0.05) compared with the control group.

The decrease in beta-globulin levels in the blood of both groups differed throughout the experiment. There was a higher content of this indicator in the blood of the experimental group compared to that in the blood of the control group (in the second study [a month before calving] by 36%, in the third study [10 days after calving] by 25%, in the fourth study [a month after calving] by 24%, and in the first study [2 months before calving] it equaled 21.4 ± 1.02).

The dynamics of changes in gamma-globulin concentrations in blood in the experimental and control groups differed significantly. Thus, 2 months before calving, the level of gamma-globulins in the blood of cows of the experimental group was 6.07 times higher than that of the control group. A month before calving, the gamma-globulin content in the blood of the experimental group increased by 26.2% compared with the value in the first study; however, an increase of 25% was observed in the control group in the same period. The arithmetic mean value of this indicator was 22% higher in the experimental group than in the control group (p ≤ 0.001).

## Discussion

The data of our study conducted at a dairy farm in the North Kazakhstan region show the influence of metabolic disorders (subclinical ketosis) on the main biochemical blood parameters of highly productive cows. Xu *et al*. [15] and Wang *et al*. [16] indicated that a comprehensive study of blood and urine morphological and biochemical parameters in subclinical ketosis is relevant. According to our results, subclinical ketosis is difficult to diagnose because there is no pronounced clinical picture, which is an important factor. The detection of this disease in highly productive cows should therefore be based solely on the results of laboratory tests of blood, urine, and milk.

Maintaining a constant internal environment is necessary for normal metabolism. The acid-base balance is one of the most important indicators characterizing the constancy of the internal environment In the course of the study, the alkaline reserve in the blood serum was determined to clarify the changes occurring in the acid–base balance of the body, because in the case of subclinical ketosis, a significant deviation in the acid–base balance can be diagnosed.

Subclinical ketosis in industrial livestock complexes showed mainly minimal symptoms. Therefore, the presence of ketone bodies and their fractions is an important indicator in the blood.

In this study, we observed an increase in ketone body concentration, with the highest levels recorded 10 days after calving. Comparing the concentration of ketone bodies in the blood of animals with subclinical ketosis before and after calving, in our study, the ketone level was 0.89 ± 0.04 mmol/L 2 months before calving, which was 25.8% lower than that reported by Gross and Bruckmaier [5] and Stevenson *et al*. [17]. A month before calving, the indicators differed slightly. In our studies, these indicators amounted to 1.0 ± 0.02 mmol/L and were 13% lower. However, 10 days after calving, these indicators increased to 1.45 ± 0.05 and 1.76 ± 0.11 mmol/L. A month after calving, there was a 26.2% decrease in the indicators in our studies at the level of ketone bodies of 1.07 ± 0.03 and 17% decrease at the level of 1.46 ± 0.08 in Gross and Bruckmaier [5] and Stevenson *et al*. [17]. There are significant differences, but the trend of decreasing and increasing indicators is the same and depends on the study period [5, 17].

Our studies showed that ketosis was detected in highly productive cows, especially in a subclinical form, because the ketone body content deviated from the average norm. The concentration ranged from 0.89 to 1.45 mmol/L, depending on the month of study in the control group, the indicator was within the normal range for all periods, whereas in the experimental group, the maximum indicator was observed in the 1^st^ weeks after calving at 1.45 ± 0.05 mmol/L. The lowest indicator was observed during the dry period 2 months before calving.

In this study, triglyceride and cholesterol levels in the blood of subclinical ketosis cows were increased compared to healthy cows. When comparing triglyceride levels, serum levels in animals 1 month before calving differed significantly (by 21.3%) from Cui *et al*. [18] and Pegolo *et al*. [19]. In our studies, the indicator was 0.61 ± 0.09 mmol/L, which was higher by 0.13 mmol/L, and the same value was found for 0.42±0.02 mmol/L. Cholesterol levels were higher than the average normal values (4.02 ± 0.21 mmol/L) and 16.7% higher than those reported by Cui *et al*. [18] and Pegolo *et al*. [19] (3.5 ± 0.22 mmol/L).

A significant decrease in the amount of glucose in the blood of sick animals was observed to be 2.00 ± 0.19. In such a case, it is impossible to convert fat into energy and ketosis develops.

One of the main findings was a significant decrease in glucose concentration in cows with subclinical ketosis, particularly 10 days after calving. This agrees with the findings of Benedet *et al*. [20] and Girma *et al*. [21]. Their studies showed that with subclinical ketosis in the experimental cows 10 days after calving, the glucose level was below normal values and amounted to 1.97 ± 0.28 mmol/L. Therefore, in our study, the glucose level in animals with metabolic disorders was the lowest for the entire period of the study at 10 days after calving. There was a decrease in glucose to 2.00 ± 0.19 mmol/L, which was lower than the minimum norm by 0.22 mmol/L. In Benedet *et al*. [20] and Girma *et al*. [21], the dynamics of changes in indicators in this period were not significant. In our previous studies by Benedet *et al*. [20] and Girma *et al*. [21], the indicator was 0.03 higher and there was no significant difference.

Comparing the blood glucose levels of sick animals with those reported by Zhang *et al*. [22] and Lean *et al*. [23], the glucose level 10 days after calving was 1.90 ± 0.15 mmol/L, which was lower than that reported by Zhang *et al*. [22] by 0.07 and 0.1 mmol/L, respectively. Comparative studies have shown that 10 days after calving with metabolic changes in animals, significant changes occur in the glucose index in the blood of animals and the indicator during this period is below the physiological limits [23].

Zhang *et al*. [22] and Lean *et al*. [23] indicated that it is impossible to maintain normal blood glucose levels because of protein gluconeogenesis. In their studies, they also pointed out that one of the causes of subclinical ketosis was an increased concentration of ketone bodies in the blood and urine of sick animals.

Previous studies [19, 24] noted that the correlation between glucose and free fatty acids is insignificant under conditions of feeding with a diet balanced according to all basic zootechnical norms. In cows with subclinical ketosis, the correlation between glucose and free fatty acids or ketone bodies in the blood is sharply negative, whereas that between ketone bodies and free fatty acids is positive [19, 24]. In our study, the same pattern can also be traced.

The findings of this study highlight the importance of monitoring and managing metabolic health in highly productive dairy cows, particularly during the perinatal period. Further research is required to explore interventions and management practices that can mitigate the risk of subclinical ketosis and other metabolic disorders in dairy cows. Identification of early biomarkers and the development of targeted nutritional strategies may contribute to improving animal welfare and profitability.

## Conclusion

In the course of our studies, we aimed to evaluate the changes in biochemical blood parameters of highly productive cows with metabolic disorders. On the basis of the obtained data, cows in the experimental group exhibited a more pronounced and intense disturbance of liver protein formation function both before and after calving compared with those of the control group. Our results suggest that the animals of the experimental group have subclinical ketosis, characterized by an increase in the number of ketone bodies and a simultaneous decrease in sugar content. According to our results, this is a consequence of a lack of available energy in metabolism with hypoglycemia characteristic of ketosis (a decrease in the amount of glucose in the body) as a result of a deficiency of starch and sugars.

A decrease in blood glucose concentration was 36% at 2 months before calving, 47.3% at 1 month before calving, and 48.3% at 10 days and 1 month after calving compared to the control (p ≤ 0.001). These indicators indicate a deficiency of carbohydrates in the body leading to an increase in the use of fatty acids as a source of energy.

The accumulation of ketone bodies in the body leads to increased neutralization of the blood buffer systems by their alkaline equivalents, which is expressed in a decrease in the indicator of alkaline blood reserves 2 months before calving by 2.79 or 14.4% relative to the control at p ≤ 0.001.

The data obtained expand and complement the scientific and theoretical provisions on the effect of subclinical ketosis on the biochemical parameters of the blood of highly productive cows and describe the correlation between changes in glucose, free fatty acids, and ketone bodies in the blood of highly productive cows.

The continuous monitoring of biochemical blood analysis in cows enables the identification of metabolic disorders at an early stage, the implementation of necessary remedial measures, the prevention of early culling of highly productive animals, the maintenance of high productivity, and the acquisition of high-quality milk.

## Authors’ Contributions

YI and MZ: Conceptualization, design, and planning of the study. DI, IS, and KN: Conducted the research, performed statistical analysis, and drafted the manuscript. DZ, BK, and BA: Sampling and delivery of samples and conducted the study. All authors have read, reviewed, and approved the final manuscript. KS and LK: Data collection and analysis and critical review of the manuscript.
